# Supplementary material for: The Impact of Male Social Status on Vaginal Secretory Responses in Mice
Source: Biology (Basel). 2025 Aug 13;14(8):1041. doi: 10.3390/biology14081041 (PMC12383819; doi:10.3390/biology14081041)
Supplement: Supplementary file 1 [file biology-14-01041-s001.zip › biology-3772101-supplementary.pdf]

Supplemental Figures

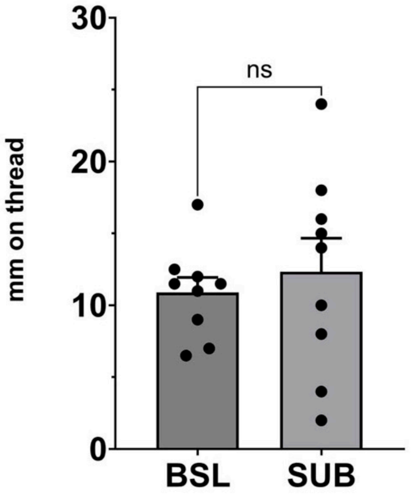

Figure S1. Vaginal secretory responses to subordinate males in females of unknown estrous phase. In mice not tested for phase of estrus, vaginal secretory responses were not seen in response to the scent of subordinate males. Measurements were taken before (baseline, BSL) and an hour after exposure to urine from subordinate males (SUB). Bar graph shows mean + SEM. ns, not significant. n=9.

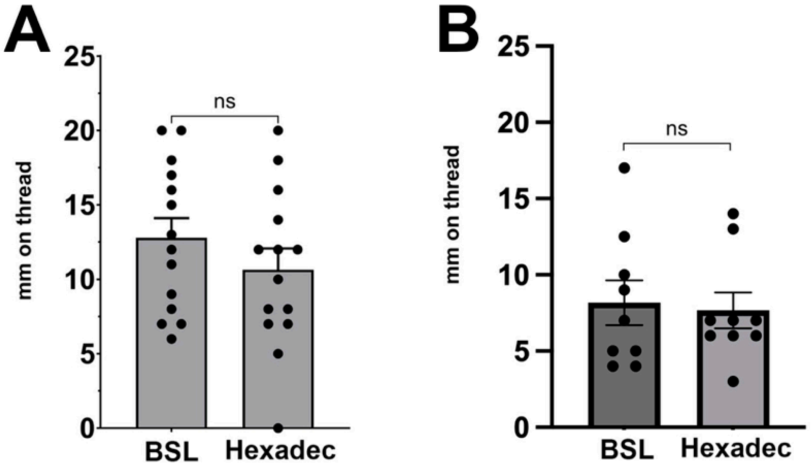

Figure S2. Vaginal secretory responses to the putative sexual attractant hexadecanol. A) In mice not tested for phase of estrus, exposure to the scent of hexadecanol ("hexadec") for 1 hour did not elicit a vaginal secretory response relative to baseline (BSL). B) Mice in their receptive phase (proestrus/estrus) allowed physical contact with hexadecanol still did not experience an increase in vaginal moisture. Bar graphs show mean + SEM. Ns, not significant. p=0.14 (A) and p=0.63 (B), by paired t-test vs. baseline, n=14, 9.
